# Increasing trees and high-albedo surfaces decreases heat impacts and mortality in Los Angeles, CA

**DOI:** 10.1007/s00484-022-02248-8

**Published:** 2022-03-24

**Authors:** Laurence S. Kalkstein, David P. Eisenman, Edith B. de Guzman, David J. Sailor

**Affiliations:** 1 Applied Climatologists, Inc, Marco Island, FL USA; 2 David Geffen School of Medicine at UCLA and UCLA Fielding School of Public Health, Los Angeles, CA USA; 3 UCLA Institute of the Environment & Sustainability, Los Angeles, CA USA; 4 School of Geographical Science and Urban Planning, Arizona State University, Tempe, AZ USA

**Keywords:** Extreme heat, Heat-related illness, Urban heat island, Urban greening, Urban cooling, Climate health

## Abstract

There is a pressing need for strategies to prevent the heat-health impacts of climate change. Cooling urban areas through adding trees and vegetation and increasing solar reflectance of roofs and pavements with higher albedo surface materials are recommended strategies for mitigating the urban heat island. We quantified how various tree cover and albedo scenarios would impact heat-related mortality, temperature, humidity, and oppressive air masses in Los Angeles, California, and quantified the number of years that climate change–induced warming could be delayed in Los Angeles if interventions were implemented. Using synoptic climatology, we used meteorological data for historical summer heat waves, classifying days into discrete air mass types. We analyzed those data against historical mortality data to determine excess heat-related mortality. We then used the Weather Research and Forecasting model to explore the effects that tree cover and albedo scenarios would have, correlating the resultant meteorological data with standardized mortality data algorithms to quantify potential reductions in mortality. We found that roughly one in four lives currently lost during heat waves could be saved. We also found that climate change–induced warming could be delayed approximately 40–70 years under business-as-usual and moderate mitigation scenarios, respectively.

## Introduction

Extreme heat is a major human health problem in urban areas, leading to many negative outcomes, including increases in emergency room visits, hospitalizations, and premature deaths. Extreme heat in cities is already the leading weather-related killer in many countries, including the USA. Annually, extreme heat causes more deaths than hurricanes, floods, and tornadoes and lightning combined; more than 7800 official heat-related deaths occurred in the USA from 1999 to 2010, and other estimates place the annual average total at 1500, with large interannual swings (Karl et al. 2009; Kalkstein et al. 2020). One analysis of 297 US counties, accounting for 61.9% of the US population in 2000, estimated an average of 5608 fatalities from heat yearly between 1997 and 2006 (Weinberger et al. 2020). A recent analysis of heat-related mortality in California found extreme heat led to 3900 deaths in California between 2010 and 2019, far more than the 599 deaths officially recorded on death certificates (Phillips et al. 2021).

Extreme heat events cause excess deaths through several pathways. Heat exposure acutely produces lethal illnesses such as heat stroke. More commonly, extreme heat exacerbates underlying, chronic conditions such as common respiratory, cardiovascular and kidney diseases. Epidemiological studies further find motor vehicle accidents, violence, suicide, and workplace injuries increase in association with extreme heat events. Thus, consecutive days of intense heat can cause dramatic spikes in incidences of a wide variety of illnesses and acute injuries, causing increases in deaths from all causes. While the health risks caused by extreme heat already pose a threat in today’s climate, the projected increases in length, frequency, and intensity of extreme heat in a changing climate loom large.

Urban areas face significant challenges as the threat of extreme heat rises, owing to a built environment that concentrates and amplifies heat, creating urban heat islands (Mohajerani et al. 2017; Li and Bou-Zeid 2013). Urban heat islands increase the daytime temperature by 4 °C (7.2 °F) and nighttime temperatures by up to 2 °C (3.6 °F) (Ortiz et al. 2018). The burden of extreme heat and urban heat islands disproportionately affects low-income urban populations (Jesdale et al. 2013). These communities often live in neighborhoods that have less urban tree cover, less vegetation, and more hardscape — living conditions which contribute to a pronounced urban heat island, and which can create a feedback loop of heating effects.

There is a pressing need for strategies for mitigating the heat-health impacts of climate change. One set of approaches includes increasing the availability of cooled public spaces (such as cooling centers) and increasing cooled homes through air conditioning or evaporative coolers (Centers for Disease Control and Prevention n.d.). Another set of mitigation strategies entails changing urban landscapes. Urban surfaces receive solar radiation, some of which is absorbed, while the remainder is reflected back into the atmosphere. Increasing solar reflectance of building roofs and walls, sidewalks, and streets with reflective coatings and materials decreases the absorption, reducing surface and ambient temperatures (Taha et al. 1988; Krayenhoff and Voogt 2010). Adding surface waters such as ponds and rivers, so-called bluespaces, also reduces urban temperatures (Broadbent et al. 2018a, 2018b). Cooling urban areas through increasing trees and vegetation is another approach to mitigating the urban heat island (Stone and Rodgers 2001; Jenerette et al. 2007). Such “green infrastructure” can be added as green roofs, parks and gardens, and street trees. The shade and enhanced evapotranspiration of green infrastructure reduces the thermal qualities of heat islands (Livesley et al. 2016; Solecki et al. 2005) and cities around the world are implementing large-scale tree planting programs to advance urban cooling (Keith et al. 2020). These approaches have been extensively studied over the past several decades (Sailor 1995, 1998; Roy et al. 2012; Santamouris 2014). A recent review of the modeling literature (Krayenhoff et al. 2021) finds that vegetation- and albedo-based urban cooling strategies can reduce urban air temperatures by 0.2–0.6 °C for each 0.10 increase in neighborhood albedo or fractional canopy cover.

This paper examines how urban land cover choices that are made at the local level can reduce heat burdens and alleviate health impacts borne by heat-vulnerable communities, using Los Angeles County as a case study. Previous modeling and observational studies have reported a wide range of cooling induced by infrastructure-based urban heat mitigation (Aleksandrowicz et al. 2017; Krayenhoff et al. 2021; Santamouris et al. 2017). Prior studies have estimated the health benefits of increasing urban albedo, but, to our knowledge, no study has compared the health impact of different combinations of increasing tree canopy and increasing albedo (Kalkstein 1991; Kalkstein et al. 2013; Jandaghian and Akbari 2021). By modeling historic heat waves and mortality data and testing the impacts that various scenarios of increased tree cover and albedo of roofs and pavements could have on reducing temperatures and heat-related death, we offer evidence that urban land cover choices can have measurable heat mitigation benefits and the potential to save many lives. We also present a first attempt at quantifying how the heat-related impacts of climate change can be delayed — or even avoided — if these land cover choices are implemented.

## Materials and methods

### Developing heat/health relationships using a synoptic climatological methodology

We first determined the historical relationships between weather and heat-related mortality for Los Angeles County. Our previous research has shown that each city reacts differently to heat in terms of the magnitude of negative health outcomes (Sheridan and Kalkstein 2004). Cities vary considerably in terms of urban structure, demographics, and climate, all of which play a role in determining their vulnerability to heat/health issues. Los Angeles County is particularly diverse across many of these categories (Table 1).

**Table 1:** Climatic and geographic diversity of Los Angeles County. Los Angeles County is unusually diverse across several relevant variables

Variable	Distinction
Geography and topography	The land area of LA County is 10,500 sq km (4000 sq mi). It is a coastal region that is both flanked and bisected by mountain ranges, with elevations ranging from sea level to 3000 m (10,000 ft) (Hall et al. 2018)
Climate	LA County includes multiple climate zones ranging from coastal, to high desert, to montane — each with its own seasonal averages of temperature and moisture. Precipitation is highly variable, with annual averages ranging from about 125 mm (5 in) in the high deserts to over 750 mm (30 in) in the mountains; the annual average for downtown Los Angeles is 375 mm (15 in) (Los Angeles County Department of Public Works 2021). LA County has a variable climate. This variability renders the region vulnerable to heat and produces excessive heat-related illnesses and deaths. Heat-related deaths occur even in the winter, when occasional heat events occur unexpectedly (Kalkstein et al. 2018)
Population	LA County has an ethnically diverse population of 10 million people that are 49% Latino, 15% Asian, and 9% Black. About 1/3 of residents are foreign born (United States Census Bureau, 2021)

The weather data (NCEI 2021) included four-times daily meteorological data, required to classify each day into an air mass category for Los Angeles. The variables include air temperature, dewpoint, cloud cover, and surface air pressure, necessary for developing the Spatial Synoptic Classification (SSC) (Sheridan 2002, 2020). The SSC has been used extensively in climate/human health analyses (Dixon et al. 2016; Hondula et al. 2014), including the development of “heat-health warning systems” around the world (Kalkstein et al. 2009), climate change–health analyses (Greene et al. 2011, 2016), and the development of a heat wave categorization system for vulnerable urban areas worldwide (Adrienne Arsht-Rockefeller Foundation Resilience Center 2020).

The SSC places each day into one of several air mass types listed in Table 2. An air mass is a volume of air defined by its homogeneous characteristics of temperature, atmospheric moisture, and other meteorological variables (Oliver and (Ed.) 2008), and research suggests that humans respond to the simultaneous impacts of numerous meteorological elements, rather than just individual weather variables (Kalkstein et al. 2018). Thus, air masses present a comprehensive picture of how organisms respond to their meteorological environment. Most frequently, two air mass types cause higher mortality when they occur throughout an urban area: dry tropical (DT) and moist tropical plus (MT +). Our study concentrated on these two, which occur relatively rarely across the Los Angeles Basin but are nevertheless responsible for the bulk of negative heat-related public health outcomes (Kalkstein et al. 2020).

**Table 2:** Summary of air mass types. Bold items indicate air mass types with statistically significant higher mortality rates

SSC air mass type abbreviation	Air mass type description
DP	Dry polar: cool, dry air mass
DM	Dry moderate: comfortable and seasonally warm
DT	**Dry tropical: hot, dry, and very oppressive**
MP	Moist polar: cool and moist, overcast
MM	Moist moderate: warmer than MP but still wet and overcast
MT	Moist tropical: typical summer air mass, warm and humid
MT + MT + +	Moist tropical **plus: excessively** hot and humid; **oppressive**
TR	Transition between different air masses; frontal boundary

Los Angeles County has three stations that provide the hourly data required to develop the SSC: Burbank Bob Hope Airport (BUR), Los Angeles International Airport (LAX), and the Marine Corps Air Station in El Toro (NZJ), which is nearby in Orange County. A fourth station, the USC downtown site, also records hourly data, but there are copious missing values, particularly overnight. We used the LAX airport station for this study because when the Los Angeles Basin is at its hottest, it is usually very hot at LAX as well despite this site being closer to the coast, owing to the surface airflow being from the east or south. All of the heat events evaluated in this study demonstrated very high temperatures and oppressive air masses throughout the Basin (Table 3). The LAX record is most complete, and all of the four extreme heat events we evaluated were intense within the entire county.

**Table 3:** Comparison of the air masses at LA Airport (LAX), Burbank (BUR), and El Toro (NZJ) weather stations. While there is some variation in the air masses, there are offensive air masses throughout the entire county for all four heat events at each of the weather stations, including LAX, which was used for this study

Data	LAX	BUR	NZJ
22-Jul-06	**MT + **	**DT**	**MT + **
23	**MT + **	**DT**	**MT + **
24	**MT + **	**MT + **	**MT + **
25	**MT + **	**MT + **	**MT + **
26	**MT + **	**MT + **	**MT + **
19-Jun-08	**MT**	**DT**	**MT**
20	**MT**	**TR**	**MT**
21	**DT**	**DT**	**DT**
22	**TR**	**TR**	**TR**
26-Aug-09	**DM**	**DT**	**DM**
27	**TR**	**DT**	**TR**
28	**MT**	**DT**	**MT**
29	**MT**	**TR**	**MT**
30	**TR**	**DT**	**TR**
26-Sep-10	**DT**	**DT**	**DT**
27	**DT**	**DT**	**DT**
28	**DT**	**DT**	**DT**
29	**MT**	**DT**	**MT**

We defined meteorological summer as the period between May 1 and September 30, and we evaluated weather/mortality relationships for all air masses during this seasonal period, hypothesizing that only the DT and MT + air masses would show statistically significant positive deviations in mortality.

Mortality data came from the Centers for Disease Control and Prevention, National Vital Statistics System (National Center for Health Statistics 2018). We examined all-cause mortality for the period of 1985–2010, which reliably estimates excess deaths during extreme heat events (Kalkstein et al. 2009; Hajat et al. 2006). We calculated the excess daily mortality value by fitting a polynomial function for average daily mortality for the summer season across the years of study, representing baseline deaths for each year, resulting in the departure from average (Sheridan et al. 2009). This approach standardizes the data adjusting for seasonal mortality cycles and any inter- or intra-annual changes in population.

We compared mortality anomalies to daily SSC air mass type to determine which air mass types are associated with increases in human mortality. We found that the DT and MT + air masses are associated with statistically significantly greater mean anomalous daily mortality above the baseline. However, not all days within these air masses demonstrate elevated mortality, so a stepwise linear regression was developed for the study area to determine which variables account for this mortality variation. The independent variables used in our analysis were meteorological (e.g., morning and afternoon temperature, dew point, windspeed, and cloud cover), persistence-oriented (consecutive days of the air mass within a particular excess heat event or EHE), and seasonal (time of season). This statistical procedure resulted in an algorithm for Los Angeles County containing statistically significant independent variables. It was utilized to estimate mortality during particular EHEs both in reality and under modeled simulations.

We then selected four specific historical EHEs to evaluate for Los Angeles County, each with unique characteristics and occurring during different times in the summer season. These were the following:

- July 22–26, 2006: a hot and humid EHE dominated by MT + air mass days
- June 19–22, 2008: a drier event with a mixture of MT + and DT days
- August 26–30, 2009: the least intense heat event of the four, enabling evaluation of a more common, less extreme EHE
- September 26–29, 2010: a very hot Santa Ana event bringing hot, dry wind from the desert, dominated by DT days

The selection of EHEs with differing air mass characteristics was a hallmark of this analysis, since each has a unique solar radiation, air quality, and wind profile signature. Thus, the synoptic approach of identifying heat events is unique and often more holistic than other methods used in the literature (e.g., Meehl and Tebaldi 2004) or by the federal government (e.g., National Oceanic and Atmospheric Administration 2021). They represent the most common types of heat events that will impact the Los Angeles Basin, and evaluating them individually permits us to determine the effectiveness of our albedo and canopy cover scenarios within each EHE type.

### County-level modeling approach

For this study, we used regional scale atmospheric modeling to simulate Los Angeles County using three nested domains with the innermost domain covering the entire county with 156 by 156 grid cells of 500 m on a side, and a total of 42 vertical levels.

The single-layer urban canopy model (Kusaka et al. 2001) was implemented using Weather Research and Forecasting (WRF) model, version 3.8.1, using default geometries for parameterized urban canyons in low density residential, high density residential, and commercial land use areas (Chen et al. 2011). The low, medium, and high intensity development categories used in the modeling are based on fraction of impervious surface as defined in the National Land Cover Database (Homer et al. 2015). Low intensity urban land cover corresponds to areas with a mix of constructed materials and vegetation, with impervious surfaces accounting for 20–49% of total cover (typically single-family housing units). The medium intensity urban land cover includes areas with 50–79% impervious surface cover (typically higher density housing). The high intensity classification is for areas with 80–100% impervious cover (typically commercial and industrial areas). Canopy layer model defaults were used to specify urban geometry and associated thermal properties. Specifically, low residential neighborhoods were assigned roof height of 5 m, with widths of roofs and roads set to 8.3 m. For high-density residential area the roof height was increased to 7.5 m with roof and road widths set to 9.4 m. More dense commercial areas were assigned roof heights and roof and road widths of 10 m. While actual urban morphology varies across and within cities, these defaults are commonly used in the modeling literature for cities in North America (Wang et al. 2011; Georgescu et al. 2014).

All simulation cases were provided atmospheric initial and boundary conditions from the National Centers for Environmental Prediction’s North American Reanalysis (NARR) 3-hourly atmospheric data. Key model physics parameterizations included the Rapid Radiative Transfer Model (RRTM) with the Dudhia shortwave radiation scheme; Monin–Obukhov (Janjic Eta) similarity scheme for the surface layer, the Mellor-Yamada-Janjic TKE scheme for boundary layer physics, and the Noah Land Surface Model. To ensure appropriate model spin-up, all simulations were conducted for a full 7 days prior to the onset of each modeled EHE. Baseline simulations were conducted for each EHE and model performance was judged by comparing hourly temperature data from multiple National Weather Service airport weather stations within the domain with model output averaged over an array of 9 grid cells centered on the weather station (e.g., 1.5 km by 1.5 km region). In general, model performance, as judged by visual inspection of the diurnal profiles, and by RMSE values (typically 2–4 °C), was good (see figures in appendix Figs. 2 and 3).

High albedo modifications were implemented by modifying the roof and pavement albedo values within the urban parameters input file. Vegetation increases were implemented by suitably modifying the urban vegetation coverage variables in the vegetation parameterization input file for WRF. We developed realistic albedo and tree cover scenarios to evaluate how these alterations might change urban meteorology and associated heat mortality totals (Table 4). Intervention scenarios were informed by a survey of relevant studies and documents pertaining to Los Angeles’ baseline UFC/albedo conditions, its efforts to increase these, and UFC/albedo increase targets adopted by cities with similar conditions (McPherson et al. 2008; City of Melbourne 2012). For the countywide analysis, a baseline number was determined to represent existing tree cover, roof albedo, and pavement albedo. Using the baseline, four scenario combinations were developed to test the relative impact of tree cover and albedo. For example, in case 1, we increased tree canopy slightly but assumed a large increase in pavement and roof albedo. In case 4, our most optimistic scenario, we increased both tree canopy and albedo to the maximum we considered feasible. For the tree canopy increases, we conducted an assessment of land cover classes by using i-Tree Canopy to identify baseline tree cover (USDA Forest Service n.d.). We used i-Tree Canopy to determine the tree cover for LA County’s urban areas only, as this is where urban populations are concentrated and where target neighborhoods would be identified for tree cover and albedo increases. We determined that existing tree cover for LA County’s urban areas is 16.6% based on 2017 imagery, with an error of + / − 1.7%. We then reviewed relevant literature and tree cover increase efforts to arrive at tree canopy increase scenarios that represented a range of ambitions, from moderate to more aggressive. To determine baseline roof and pavement albedo and produce scenarios for their increase, we reviewed relevant literature and efforts and relied on Lawrence Berkeley National Laboratory’s Hot Roofs, Cool Roofs mapping tool, based on 2009 imagery (Lawrence Berkeley National Laboratory n.d.). Through this process, we arrived at the estimate that the existing albedo for LA County was 17% for roofs and 10% for pavement.

**Table 4:**
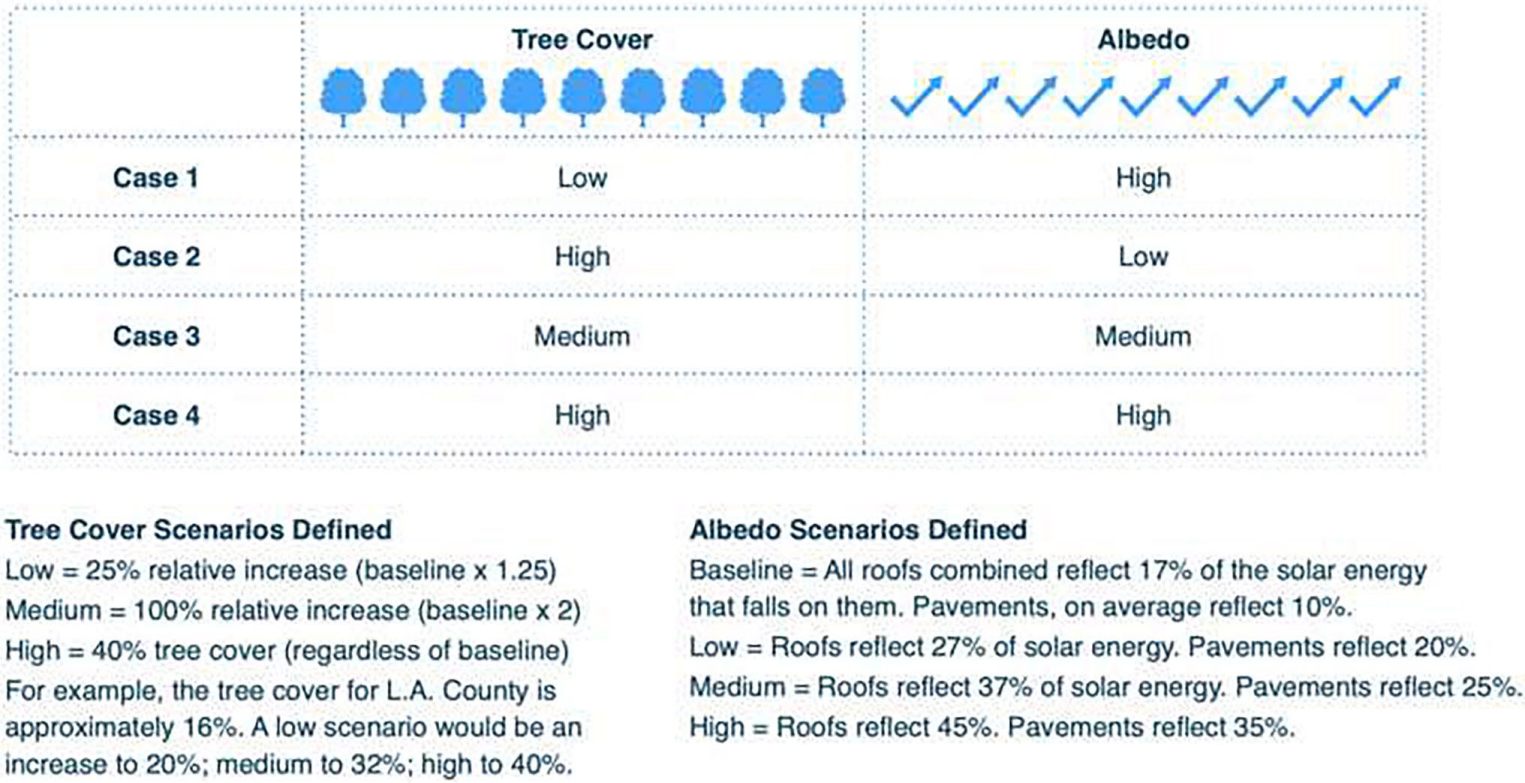
Albedo and canopy cover scenarios used in this research. The control case was used as a baseline to be closest to present conditions in Los Angeles County

## Results

### Mortality algorithm development

Correlating our mortality data with the meteorological data for the offensive air masses (DT, or dry tropical, MT + , or moist tropical +), we arrived at the following algorithm for Los Angeles County utilizing only statistically significant variables at the *p* < 0.05 level:

$$\% MORT = -1.426 + 0.363 NFPTS + 5.219 DT + 1.609 MT + 0.057 AT05$$

where % MORT is the percent change in mortality from the baseline value (we consider this heat-related mortality), NFPTS is the Nairn-Fawcett Extreme Heat Factor (Nairn and Fawcett 2014), which evaluates heat in three consecutive day increments and determines whether the period before the heat wave has been hot or comfortable (which could have a significant difference on response), DT is a dummy variable which is added just for the DT air mass days, which is a highly transparent air mass with minimum cloud cover and significant solar radiation income, MT is a dummy variable for MT + which is added just for the MT + days, and AT05 is 5AM apparent temperature. Utilizing this algorithm, which is based upon DT and MT + heat events spanning the period 1985–2010, we noted that during an average 5-day Los Angeles County EHE during this timeframe, excess mortality is 4.1% on the first day of the event. In the case of a 5-day heat event, like two of the EHEs we evaluated, it increases to 11.9% on the fifth day of the event.

This mortality algorithm was applied to the baseline (or control) meteorological conditions during the four evaluated heat events, as well as the four cases of mitigation scenarios presented in Table 4.

### Meteorological and health impacts of the mitigation scenarios

We saw clear changes in air temperature and dewpoint temperature across all four heat events utilizing the four mitigation scenarios, consistent with studies conducted in other cities (Kalkstein et al. 2020). Temperatures mostly showed decreases in the range of 1–2 °C (1.8–3.6 °F; Table 5 and 6), while dewpoint temperatures showed similar increases in magnitude. Cases 1 and 3, which have more modest urban tree cover increases than the other scenarios, show smaller changes than cases 2 and 4 with the most aggressive tree canopy increases. This is intuitive, since added tree cover would add water vapor into the atmosphere through evapotranspiration, thus increasing the dewpoint temperature. Nevertheless, in general, the largest decreases in temperature also occur in cases 2 and 4, sometimes exceeding 2.5 °C (4.5 °F), especially during the August 2009 event (Table 6), when maximum temperatures reached 36–40 °C (97–104 °F), depending on the location.

**Table 5:**
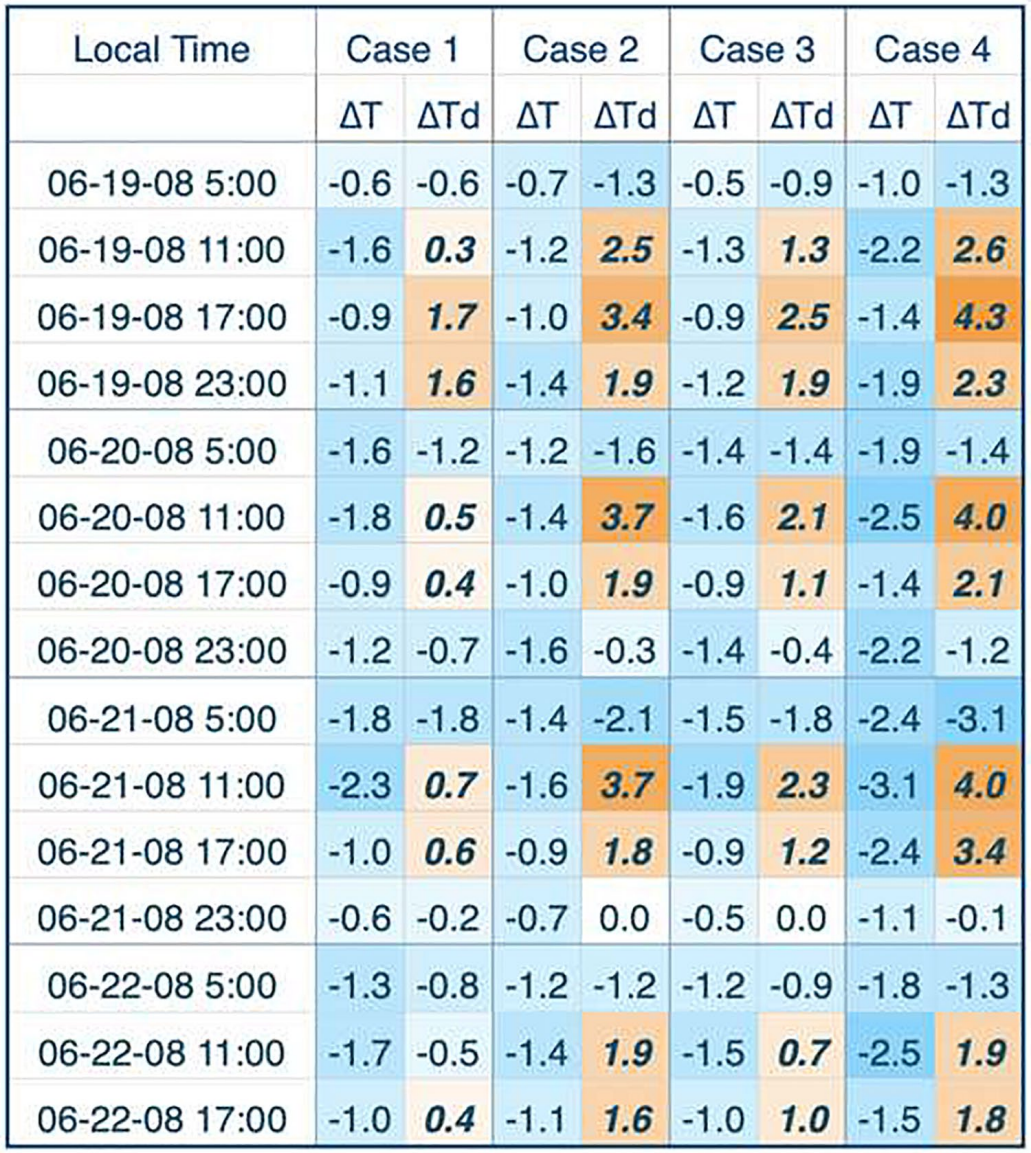
Changes in meteorology for the June 2008 heat wave. All four scenario cases are presented. Delta T is the change in temperature (°C) from the baseline. Delta Td is the change in dewpoint temperature (°C) from the baseline. Increasingly dark (blue) color represents greater reductions; increasingly dark (orange) color in bold italics represents greater increases

**Table 6:**
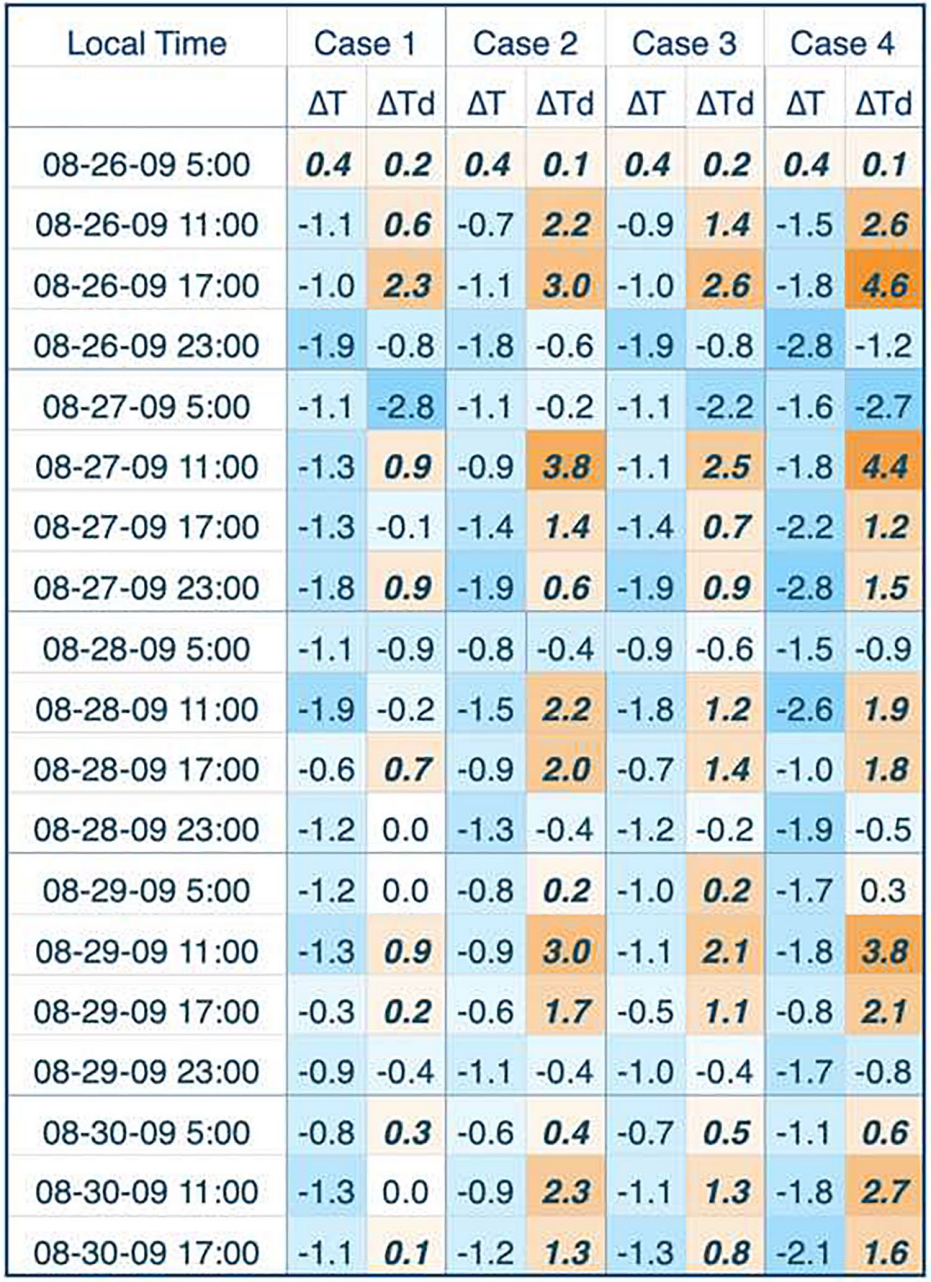
Changes in meteorology for the August 2009 heat wave

Besides evapotranspiration increases, some of the increases in dewpoint temperature are physically attributed to the cooling temperatures themselves, especially for cases 2 and 4. When temperatures are cooled, vertical motion of the atmosphere is inhibited, and the dispersal of near-surface moisture is therefore less efficient. Thus, moisture from sources such as car exhaust, air conditioning, and even from trees is less likely to be dispersed vertically and more likely to accumulate near the ground.

However, the decrease in air temperature is more important in terms of human well-being than the accompanying increase in dewpoint temperature. The *apparent temperature* (National Oceanic and Atmospheric Administration 2021), which is the perceived temperature by humans and represents the combined impacts of thermal and moisture characteristics in the atmosphere (sometimes called the “heat index”) is impacted more by a drop in temperature than a rise in dewpoint temperature. For example, an air temperature of 40 °C (104 °F), coupled with a dewpoint temperature of 20 °C (68 °F) yields an apparent temperature of 44 °C (111 °F). If the temperature is dropped to 37 °C (99 °F) and the dewpoint temperature is raised to 22 °C (72 °F) — something that is common within the scenarios we modeled for this study — the apparent temperature drops to 42 °C (107 °F). Thus, the air temperature plays a more important role in human perceived conditions than does dewpoint temperature.

The impact of these thermal changes has a significant effect upon human mortality, as indicated by our modeling (Table 7 and 8). The June 2008 EHE provides a good example (Table 7), and mortality increases above the baseline are reduced for each scenario, particularly case 4. As the heat event continues, the mortality increase becomes greater; for the baseline, the percentage increase is 1.2% for the first day of the event to 13.5% for the fourth consecutive day, a common result for all of our modeling using the aforementioned mortality algorithm. On average, approximately 150 people die daily during summer in Los Angeles County from all causes (Los Angeles County Department of Public Health 2015). Thus, a 13.5% increase in mortality represents about 20 extra deaths from heat on June 22, 2008, an unfortunate and considerable number of deaths. If we sum the percentage increases for all 4 days of the EHE, the mean 6.9% increase represents about 41 extra deaths which we relate to heat during the entire event (6.9% of 600 total deaths equals 41 excess deaths during the 4-day heat event). For June 19–20, 2008, the baseline indicates that an MT air mass was present for those days. There was an air mass change to DT on June 21, while a transition air mass (a change from one air mass to the next; indicates a cold front passage) was present on June 22.

**Table 7:**
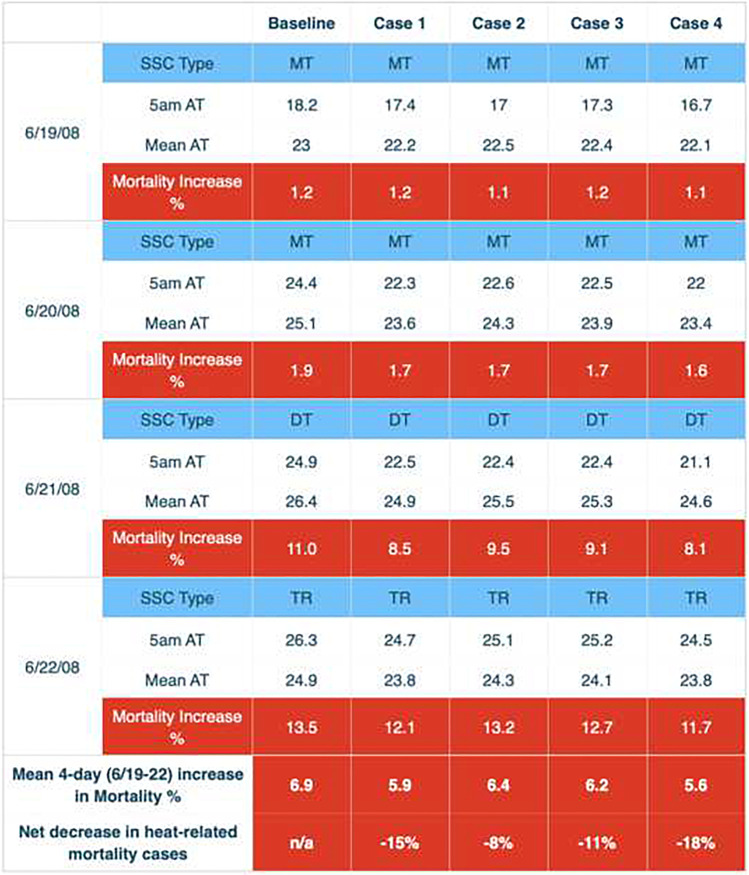
Changes in air mass type, apparent temperature (AT), and mortality for June 2008 EHE. 5AM and mean daily apparent temperature are displayed for each day during the EHE. “Increase in mortality %” represents percent increase in excess mortality over the daily mortality standardized value. The mean increase for all the EHE days is shown at the second row from the bottom. The net decrease in heat-related mortality from the baseline is shown in the bottom row. “SSC Type” shows air mass type; darker cells in bold italics show actual changes in air mass type due to a significant meteorological change

**Table 8:**
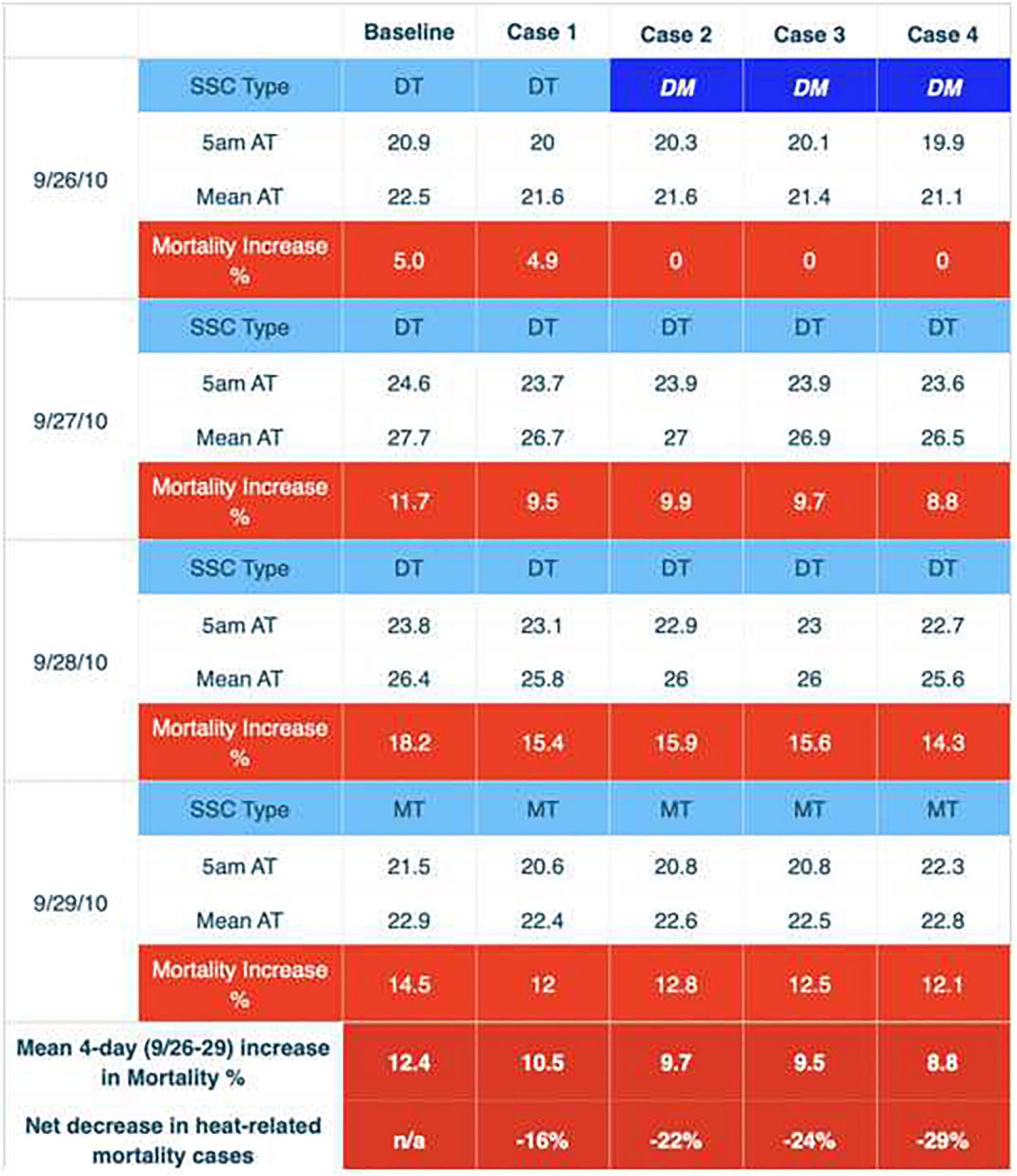
Changes in air mass type, apparent temperature (AT), and mortality for September 2010 EHE

For case 1 on June 19, there was no reduction in excess mortality although apparent temperatures were somewhat lower (1.2% above the baseline). Yet, reductions can be seen for the other 3 days of the heat wave: on the 20th, from 1.9% in the baseline to 1.7%; on the 21st, from 11% in the baseline to 8.5%; and on the 22nd, from 13.5% in the baseline to 12.1%. Thus, for the entire 4-day heat event period, case 1 produced a 1 percentage point decline in excess mortality, from 6.9 to 5.9%. This is a 15% decrease in heat-related mortality, and represents about 6 saved lives (from 41 excess deaths to 35 deaths). In contrast, case 2 only reduced excess mortality by 8% (6.9–6.4%) when compared to the baseline. Case 3 did slightly better than case 2, but case 4, the most aggressive case in terms of increasing tree cover and albedo, reduced excess mortality by 18%, or about 8 deaths (from 41 to 33). We find these results to be encouraging, as they indicate that heat-related deaths could be reduced significantly in a heat event of this type.

The September 2010 event (Table 8), a dry Santa Ana situation, had even more dramatic outcomes. Most of the days during this EHE were DT (dry tropical), the air mass type that kills the most people in Los Angeles. On September 26, the beginning of the heat event, there was an actual air mass change under cases 2, 3, and 4, from DT to a more benign dry moderate (DM) air mass. Such air mass changes are rare in similar evaluations of cities. This change has a great impact on reducing heat-related mortality, as can be seen for cases 2, 3, and 4 on September 26, where no heat-related mortality was estimated. During this EHE, the mean percentage reduction on the days when excess mortality was estimated dropped by 29% for case 4 (8.8% is a 29% reduction from 12.4%), which is the equivalent of saving 23 lives during that heat event (from 78, based on our algorithm for this heat event, to 55 deaths). This result was among the most encouraging we have seen for such heat wave analysis in any large urban area.

The July 2006 heat wave was hot and humid, showing some monsoonal influence with MT + air masses dominating. Temperatures on the 22nd and 23rd approached 38 °C (100 °F) and dewpoints were near 16 °C (61 °F). The results were somewhat similar to the two previous heat waves evaluated: the temperature decreases under the four scenarios were rather significant, with decreases ranging generally from 0.5 to 2 °C (0.9–3.6 °F) for cases 1, 2, and 3, and generally 0.5 to 1.0 °C (0.9–1.8 °F) lower than that for case 4. Dewpoint simulations showed similar increases to other heat waves in the analysis, varying from slight decreases to increases up to 2 °C (3.6 °F). Notably, the greatest temperature decreases and dewpoint increases were observed at night, when thermal changes often have the greatest impact on human health.

Three of the 5 days during this heat event demonstrated some air mass change. For July 22 and 26, all four cases resulted in changes from MT + to MT, a more benign air mass. On July 25, we saw a change from MT + to MT for case 4, our most aggressive scenario. Mortality percentages diminished, particularly for case 4. In fact, the decreases in excess mortality range from 9% for case 2 to 18% for case 4, certainly significant decreases attributed to the modeled alterations in albedo and vegetation.

The August 2009 heat event was somewhat drier; unlike the 2006 event, there were some DT and DM days and dewpoint temperatures were generally below 10 °C (50 °F). As is often the case when drier heat events are present, the results were more variable than the more humid events. Temperature decreases usually exceeded 1.0 °C (1.8 °F), and frequently approached 2.0 °C (3.6 °F), especially for case 4. In this heat wave, we saw instances that exceeded a 3 °C (5.4 °F) decrease, which is especially large. Drier air masses possess a lower specific heat than more moist air masses, which permits them to gain or lose energy at a faster rate. Thus, it is possible to see these more extreme results, with greater daily swings. This is particularly the case for dewpoint temperature, which shows up to an almost 8 °C (14.4 °F) swing between increases and decreases during the heat event. We have closely examined these large dewpoint swings, and there is nothing that we observed to consider that these are not feasible, based upon the scenarios and modeling that we used. However, we think that results from this August 2009 heat event should be observed with greater caution than the other EHEs because of the high variability in the results.

For the 2009 event, we also saw some air mass changes. Three MT days changed to DM, a generally cooler and more comfortable air mass. There were also decreases in excess mortality percentage, but the increases in mortality from the heat were much smaller for this event than for the other three. Temperatures for this event were the lowest for all four heat waves evaluated, hence the lower mortality increases. Thus, the percentage decreases, though very large, are to be regarded with some perspective, noting that overall heat deaths were lower.

### Local effects on climate change

The results of this study suggest that we have existing technologies to significantly lessen the impacts of heat on negative health outcomes. However, there are also some important climate change implications that arise from our analysis.

**Fig. 1:**
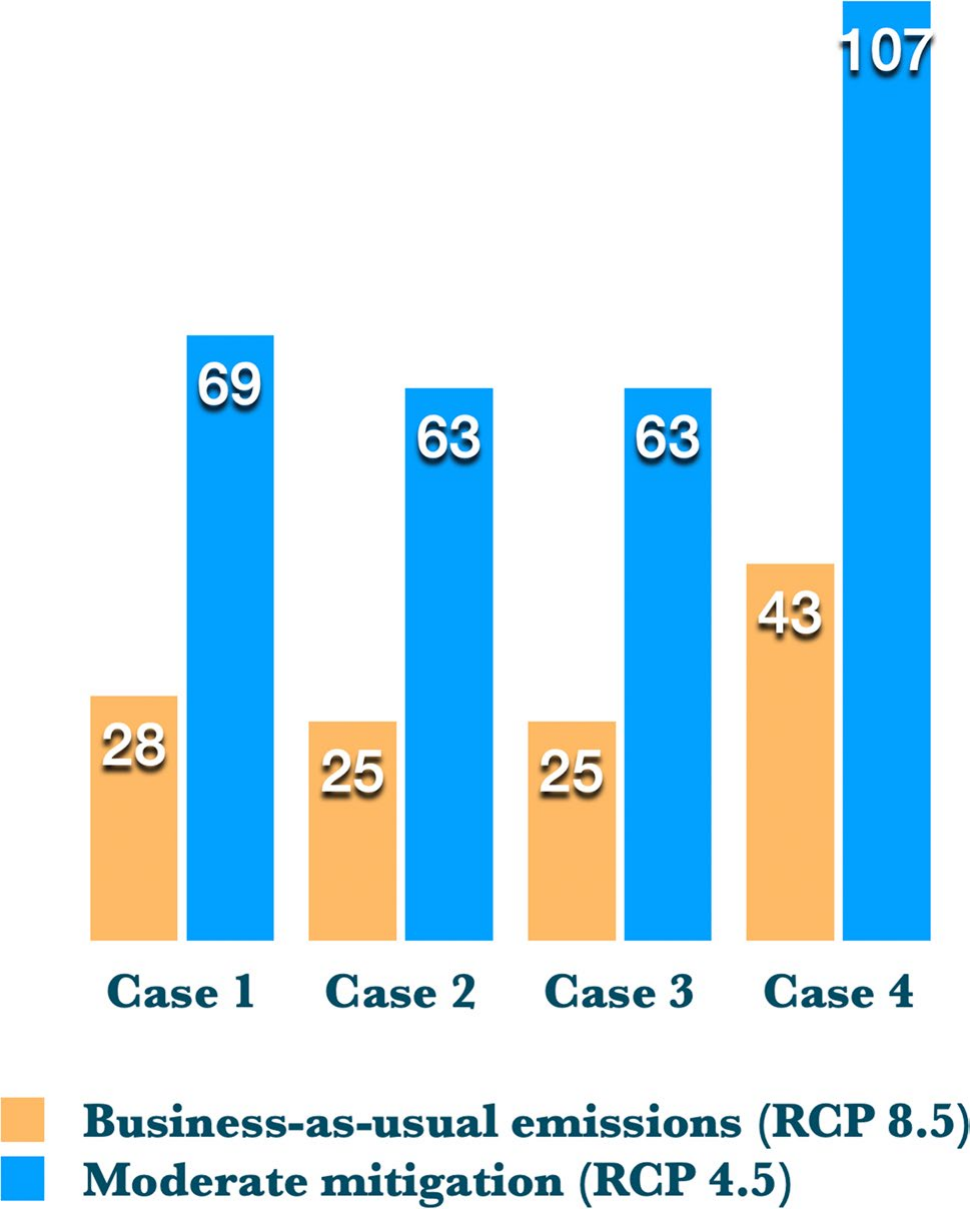
Years of delay of climate change–induced warming under the four albedo/canopy cover scenarios. Bars indicate the number of years of delay that would result from each case under either RCP 8.5 or 4.5

A typical approach to evaluating the impact of climate change upon excess mortality is to apply climate models to mortality algorithms and determine how many additional deaths would occur under the various emissions scenarios (e.g., Sheridan et al. 2012). In this study, we departed from the approach and attempted something quite different. Rather, we estimated how many years of climate change–induced warming we could potentially delay if the four albedo/canopy cover scenarios offered in this evaluation were implemented. We modeled the four cases under both business-as-usual and moderate mitigation scenarios at the Los Angeles County level by utilizing the Representative Concentration Pathways (RCP) models 8.5 and 4.5 (van Vuuren et al. 2011; Schwalm et al. 2020), approved by the Intergovernmental Panel on Climate Change (IPCC). The results are intuitive and allow us to accept our hypothesis that these cooling scenarios can potentially delay climate change–induced warming in Los Angeles by a number of years or even decades (Fig. 1).

To accomplish this, we initially determined the mean reductions in maximum temperature for Los Angeles County, using the same tree cover/albedo prescriptions that we used in the rest of the study. The mean reduction for each case is 1.09 °C (1.8°F) for case 1, slightly less than 1 °C for cases 2 and 3, and a considerably larger 1.71 °C (3 °F) for case 4. We examined the 90th percentile of daily maximum temperature for the entire year and then just for summer (May through September). Using modeled data for the years 1950–2099, the average temperature increases under the business-as-usual and moderate mitigation scenarios were + 0.039 °C and + 0.016 °C per year, respectively. We then divided the average temperature reduction of the four tree cover and albedo cases by those average annual temperature increases to determine how many years of warming could be delayed. For example, implementing case 4 (high tree cover and high albedo) would reduce temperatures by an average 1.7 °C, so we find that 1.7 / 0.039 = 43 years of possible delay. This means that climate change–caused warming could be potentially delayed approximately 43 years relative to a business-as-usual emissions scenario (RCP8.5) if tree cover and albedo were to be increased aggressively. In this example, Angelenos could experience a climate in the year 2063 that was like the climate in year 2020. For the moderate mitigation scenario (RCP4.5), the delays would be greater, related to the lesser slope of temperature increase in that model. Assuming the case 1 example above, the 1.09 °C (nearly 2 °F) decrease would delay the warming by 69 years (1.09/0.016), since the slope of temperature increase for the RCP4.5 model is less than half of RCP8.5. Thus, if we could meet the emissions demands of RCP4.5, the effectiveness of the cooling will yield an even greater delay in climate change–caused warming. We believe this is a novel way to estimate how urban cooling scenarios based upon presently-available technologies can potentially mitigate climate change–induced warming effects in urban areas.

## Discussion

This study demonstrates that at present, we likely have the capability to cool urban areas sufficiently to change local meteorology during extreme heat events, and this cooling can lead to significant decreases in mortality during heat events. For the first time, we have provided estimates on how many lives can be saved if certain urban modifications are made to cool cities. This research suggests that this number can exceed 25% lives saved during extreme heat events if aggressive action to increase urban reflectivity and add tree canopy is taken. Temperatures during these heat events can be decreased by 2–3 °C during the hottest times of day and even overnight. Heat events will still exist, but the magnitude of the heat can be reduced to below-lethal thresholds for many individuals. That is the goal to be achieved, and it is realistic with present products and strategies, including reflective roofing and road materials, and increased tree canopy.

Our results align with prior studies estimating the reduction in heat-related mortality accruing from urban infrastructure-based mitigations. Examining two historical heat waves in Toronto and Montreal, Canada, Jandaghian and Akbari (2021) estimated a 3–7% mortality reduction from increasing surface albedo, equivalent to 7 to 18 lives that would have been saved in each heat event. Similarly, increasing surface albedo changed the air mass type in Detroit, Los Angeles, New Orleans, and Philadelphia providing an average 5–10% reduction in heat-related deaths (Kalkstein et al. 2013). Another study focused on historical extreme heat events in Los Angeles tracked the estimated temperature and dew point conditions experienced by three population subgroups—elderly, office workers, and outdoor workers—in both indoor and outdoor settings (Sailor et al. 2021). That study explored the same four levels of vegetation- and albedo-based heat mitigation strategies, finding that mitigation efforts similar to those explored in the present paper can reduce air temperature of outdoor and unconditioned indoor environments by 1–2 °C (1.8–3.6 °F), and that such reductions resulted in a decrease of excessive heat hours (a measure of total heat exposure) by 24–40%. This level of reduction in exposure is comparable in magnitude to the reduction in heat mortality found in the present study.

Using research to encourage heat intervention raises some key questions. How does a city or county make decisions about where people are most vulnerable to extreme heat? How do decision-makers determine where to concentrate resources to maximize reductions in heat-related morbidity and mortality? How can relevant government agencies and nonprofit organizations work together with community groups, academia, and residents to select and implement mitigation strategies that will work to cool neighborhoods and also fit culturally with community needs and desires? The methodology outlined in this research, and plans for subsequent related projects, describe a process to effectively engage public–private-academic partnerships to produce the data necessary for community leaders and government agencies to work with residents in a collaborative and data-driven fashion. The data from this study have already helped to inform local and state heat mitigation planning. In a separate but related analysis, LAUCC has also pursued a research agenda to divide Los Angeles County into socially homogeneous “districts” to see the differential health responses to heat among a range of communities that vary in income, ethnicity, and housing density. These results will be presented within a subsequent manuscript. The implementation of reflective materials and additional tree canopy within these diverse local regions has suggested that certain heat-vulnerable areas in Los Angeles may benefit more from such urban structural modifications. These results are enlightening and have the potential to inform heat mitigation actions at the city and neighborhood level.

## Figures and Tables

**Fig. 2:**
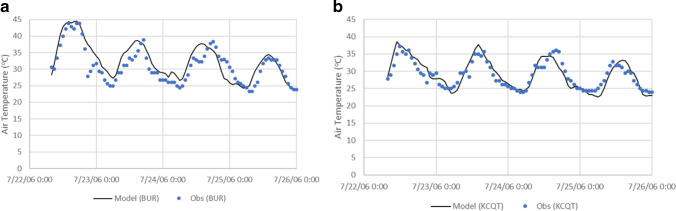
Root mean square error (RMSE) for the July 2006 Excess Heat Event (EHE). (**a**) RMSE July 2006 EHE for BUR and (**b**) RMSE July 2006 EHE for KCQT. Simulated versus observed air temperature shows the model performance (measured by RMSE) is 2.9 and 2.0 °C for Burbank and KCQT weather stations, respectively

**Fig. 3:**
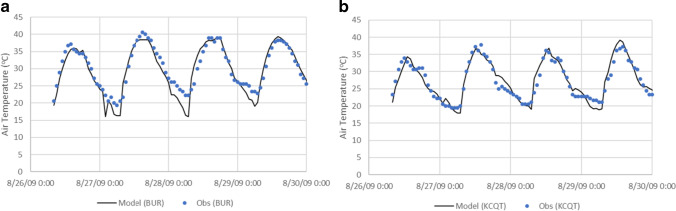
Root mean square error (RMSE) for the August 2009  Excess Heat Event (EHE). (**a**) RMSE Aug 2009 EHE for BUR and (**b**) RMSE Aug 2009 EHE for  KCQT. Simulated versus observed air temperature shows model performance (measured by RMSE) is 2.4 and 2.0 °C for Burbank and KCQT weather stations, respectively

## Data Availability

Data tables other than those presented in the manuscript will be made available upon request to the corresponding author.

## Appendix

Figs. 2 and 3
